# Readiness or Impairment: Cognitive and Linguistic Differences Between Children Who Learn to Read and Those Who Exhibit Difficulties With Reading in Kindergarten Compared to Their Achievements at the End of First Grade

**DOI:** 10.3389/fpsyg.2021.614996

**Published:** 2021-02-25

**Authors:** Ariel Ne'eman, Shelley Shaul

**Affiliations:** Edmond J. Safra Brain Research Center for the Studies of Learning Disabilities, Department of Learning Disabilities, University of Haifa, Haifa, Israel

**Keywords:** learning how to read, kindergarten, cognitive abilities, linguistic abilities, school readiness, development and maturation

## Abstract

Many studies have attempted to identify measures that predict reading abilities. The results of these studies may be inclined to over-identification of children considered at risk in kindergarten but who achieve parity in reading by the end of first grade. Therefore, the current study sought to analyze the specific cognitive and linguistic predictors of reading accuracy and reading speed separately. Additionally, the study examined if it is possible to use empirically validated measures to distinguish between children who are not ready to learn how to read in kindergarten but manage to acquire reading skills by the end of first grade, and those who continue to exhibit difficulties. The study followed 98 kindergarteners (43 boys and 55 girls) aged 4 years 10 months to six years from three different schools, who were taught how to read in kindergarten. Multiple measures of general cognitive skills, linguistic abilities, and reading abilities were measured at three different points in time: the beginning of kindergarten, the end of kindergarten, and the end of first grade. The study found that most of the children with good literacy and cognitive abilities learned how to read by the end of kindergarten. The analysis revealed a significant difference in cognitive abilities, such as executive functions and memory, which distinguished between the ability to acquire fluent reading and accurate reading. The study was able to successfully distinguish between “children with difficulties” and “un-ready” children. These results have various implications, especially in regard to the identification of and intervention with kindergarten children at risk for reading disabilities.

## Introduction

Reading acquisition is a complex cognitive process that incorporates various measures including linguistic skills such as vocabulary, syntax, and phonological awareness (Adams, 1990; Primor et al., 2011); cognitive abilities such as visual perception, memory (Gvion and Friedmann, 2004), letter naming (Kirby et al., 2010); executive functions (McClelland et al., 2007) and speed of processing (Breznitz, 2006, 2008). Several domains have been found to predict success in reading acquisition such as literacy knowledge, rapid automatized naming (RAN), verbal memory, and executive functions (EF) (Poulsen et al., 2017). The question is, which measures are best capable of predicting reading abilities in the most valid way?

Predicting success in reading acquisition is important because it allows for an improvement in early identification of children at risk for future difficulties. Accurate predictors also provide an opportunity to implement appropriate interventions necessary for remediation. There is a high likelihood that children with initial reading difficulties will continue to struggle with reading (Juel, 1988; Lonigan et al., 2000). Various studies have illuminated the significance of early detection of children with difficulties and the considerable benefit of early intervention to those students (Partanen and Siegel, 2014; Simmons et al., 2014).

Previous studies found that different abilities can predict reading success. For instance, kindergarten measures of early literacy indicators, including phonological awareness, rapid naming, and basic cognitive skills like memory, are good predictors of reading success in later grades (Shatil, 1995; Caravolas et al., 2012; Carroll et al., 2014; Partanen and Siegel, 2014). However, there is a concern that previously studied measures are inclined to over-identify such that some children who achieve low scores on the various measures will eventually acquire adequate reading abilities.

One of the basic problems in predicting future reading abilities is that screenings in kindergarten may not be accurate (Catts et al., 2009, 2015; Compton et al., 2010). Some children who have passing scores on initial screenings later experience difficulties. In contrast, other children have failing scores on the initial screenings, but are still able to achieve reading success over time. Therefore, additional variables, such as rate of knowledge acquisition and changes in development (maturation), must be taken into account in order to more accurately recognize children at risk (Torppa et al., 2019).

Few studies have investigated what distinguishes children who improve their reading abilities, compared to those who continue to have difficulty in reading acquisition (Fletcher et al., 2018). The aim of the current longitudinal study is to follow children from the beginning of kindergarten, when they start to learn how to read, until the end of the first grade and to explore the specific cognitive and linguistic predictors of reading accuracy and reading speed separately, to examine whether it is possible to use these measures to distinguish between children who are not ready to learn how to read in kindergarten but manage to acquire parity in reading skills by the end of first grade, and those who continue to exhibit difficulties.

### Linguistic Factors in Reading Prediction

There is no doubt that linguistic abilities play a key role in predicting reading acquisition. These contributions have been found to be significant in many studies and in many languages. Early literacy skills are the foundations on which reading and writing abilities develop. These language and literacy skills, include knowledge and attitudes toward reading and writing, and develop before formal instruction begins (Snel et al., 2016). Previous studies identified letter knowledge, that is recognition of letters by name (naming ability) and/or by sound, to have predictive power of reading success (Schatschneider et al., 2004). In addition, a longitudinal study exploring the prediction of reading abilities from age three to 16, found that semantic ability and interest in books at age three, as well as phonological awareness at age six, best predicted reading abilities at 16 (Frost et al., 2005).

Phonological awareness is one of the strongest predictors of reading success (Ehri et al., 2001). Previous studies show that phonological awareness is a critical component of acquiring early decoding skills (Brady et al., 1994). Previous studies have found phonological awareness to be a strong, consistent, and key predictor of a child's success or failure of reading acquisition. The relationship between phonological awareness and reading success has also been demonstrated in many studies and across several different languages (Badian, 2001; Kirby et al., 2003; Frijters et al., 2011; Norton and Wolf, 2012; Warmington and Hulme, 2012). Partanen and Siegel (2014) conducted a longitudinal study that examined which preschool measures best predicted reading acquisition in seventh grade. The study found that the best predictors of successful reading acquisition are phonological memory, knowledge of letters, and RAN. In the current study, we examined the linguistic profiles of children with different reading abilities (accuracy and fluency) and the role of linguistic abilities in distinguishing between children who are not ready to learn how to read, but will learn how to read with maturation and children with true difficulties in reading acquisition.

### Cognitive Factors in Reading Prediction

In addition to the linguistic factors discussed above, various cognitive abilities such as RAN and memory have also been found to have good predictive power for successful reading acquisition. Several studies found that difficulties with RAN predicted reading difficulties (Wolf and Bowers, 1999; Schatschneider and Torgesen, 2004). RAN is a particularly strong predictor of reading fluency and word identification. Additionally, RAN has been found to predict success with non-word reading, phonological awareness, and verbal-visual associative links (Warmington and Hulme, 2012). Other studies have shown that working memory (WM) is very important for reading success, too. WM is an efficient predictor of reading abilities. Research has found that children and adults with reading difficulties were found to have lower working memory abilities., In fact, a significant difference in performance on working memory tasks was found between preschool children who subsequently struggled with reading and their grade normal peers (Nevo and Breznitz, 2011, 2013; Partanen and Siegel, 2014). Additional factors found to predict reading acquisition are related to different types of visual processing. In their longitudinal study, Meyler and Breznitz (1998) found that verbal and visual memory significantly predicted encoding abilities. Short-term visual memory was an especially strong predictor in Hebrew. Recent research has also found a strong connection between visual processing skills and reading acquisition. Therefore, visual abilities may be a crucial component in creating the orthographic knowledge needed for reading (Bosse et al., 2013).

In addition to the cognitive factors mentioned above, executive functions (EFs) play a key role in reading success. EFs are capable of predicting future academic achievements, including reading acquisition. Studies have shown a correlation between a child's ability to regulate his/her behavior and thoughts, and acquiring academic skills such as decoding, spelling, and numeracy. EFs have also been linked with pre-academic skills such as early literacy and mathematical knowledge (McClelland et al., 2007). EFs were also found to be less efficient among children with dyslexia compared to typical readers (Reiter et al., 2005). Finally, EFs, particularly inhibition, are associated with working memory, which were jointly found to be associated with low reading achievement among dyslexic readers from childhood through adulthood (Chiappe et al., 2000). In contrast, other studies suggest that the different domains that predict reading success change with time, with age, and with exposure to written text. For instance, Ellis and Large (1988) found that each stage of development has its own cognitive components. They further discovered that the first stages of reading development rely predominantly on the visual components, which predict success in reading. In contrast, later stages are characterized by the development of phonological knowledge and components of auditory memory. Therefore, these components play a central role in predicting success at later stages.

### The Issue of Predictive Validity

Despite all of the above, different studies on predictors of reading acquisition appear to consider a child's lack of success to be an indicator of possible risk of reading disability. However, the lack of success may actually stem from un-readiness and/or a lack of sufficient exposure to a given measure. Indeed, Shatil (1995) found that it is possible to successfully predict reading abilities in a certain percentage of children, while the predictions were less successful for the rest of the children studied. This may be because some of the children were not ready to learn to read when the test was administered or had never been exposed to the type of measures employed by the investigators. Therefore, naïve participants had little success despite subsequent mastery of reading.

Moreover, a meta-analysis conducted by the NELP (National Early Literacy Panel; Lonigan and Shanahan, 2009) explored the effect of age and time of measurement on the different predictive domains. Differences were found for various measures such as phonological memory and visual perception, by age and by time of measurement. These findings show that the longer the interval between the first and second measurements, the greater the level of exposure. That is the children have more experience which effects the various measures and their correlations. As a result of this, the findings of the various studies in the meta-analysis were divided into two parts: studies on preschool children up to age 4, and a second group of studies with kindergarteners. Most of the predictive measures were not affected by age and remained constant across the two groups of studies. In three measures age had a considerable effect. Short-term phonological memory and visual perception had strong predictive power of encoding abilities, when measured in younger ages in contrast to the studies on kindergarteners. In contrast, literacy knowledge, including the child's ability to write his or her name, had a high prediction power when measured in kindergarten, but not in studies that measured this ability at younger ages (Lonigan and Shanahan, 2009).

The emphasis on a child's age, experience, and exposure to a language's writing system, and to learning reading, was also evident in the study conducted by Speece (2005). She addressed various studies on predictive measures and their limitations. Some of the limitations were characterized by under-identification (children who were eventually found to have reading difficulties but were not identified in kindergarten) and over-identification (wherein children identified as being at risk were eventually found to have adequate reading skills). A possible explanation for the misidentification is that children continue to develop in the tasks investigated by the tests. Therefore, it is difficult for these tests to predict reading ability. Moreover, some tests seem to have a floor effect, that is the tasks are very difficult due to the children's young age and inexperience. It may be problematic to assume that these children will have future difficulties with reading based on the results of those measures. In order to overcome this lacuna and to include the variables of exposure and experience to the measures, Speece suggested including another measure in the prediction, the learning measure. This proposal is in line with the Response to Intervention (RTI) model. According to this model, children who respond well to intervention will proceed on an adequate reading course despite low scores on initial measures. In contrast, children who do not respond well to intervention will probably continue to struggle in their attempts to learn how to read, and subsequently struggle in various stages of reading to learn. This study indicates that reading intervention contributes to improving the level of reading, despite a child's low initial scores. Moreover, the measured skills can improve as a result of intervention. That is, a given status at a certain age, or at a certain level, does not necessarily predict future achievement. This was supported by various studies showing that exposure and practice can affect the different measures. For instance, RAN ability can improve (Fugate, 1997; Conrad and Levy, 2011; Ne'eman and Breznitz, 2012) over time. Memory may also improve following practice (Holmes et al., 2009).

### Prediction of Reading Improvement

A learning affect does seem to exist, though the impact of this improvement on reading is not entirely clear (Holmes et al., 2009; Kirby et al., 2010). The possible improvement in children's performance after (instruction/intervention/time) was discovered by Phillips et al. (2002). Their study found that of the children defined as ‘below average’ at the end of first grade, 50% were reading at the average level in sixth grade. This and other studies (Cunningham and Stanovich, 1997) suggest that some children are able to catch up with their peers despite an initial gap.

A number of studies have investigated which abilities help children to improve their reading skills. Several factors were found to predict improvement such as vocabulary, home literacy environment, RAN, phonological awareness, verbal memory, intelligence, and classroom behavior (Scarborough, 1998; Torgesen et al., 1999; Torgesen, 2000; Fletcher et al., 2011; Denton et al., 2013). Torgesen and Davis (1996), also attempted to predict reading improvement according to participant's response to intervention. Their study examined preschoolers whose scores on a phonological awareness measure were below the 20th percentile after participating in a phonological awareness intervention. The results revealed that the measures of RAN, verbal ability, and writing nonsense words best predicted which children's reading would improve. A study with German children found that phonological memory was the best predictor of reading improvement (Schneider et al., 1999). Torgesen et al. (1999) examined the impact of three intervention programs on children who scored in the lowest percentiles (12th percentile or lower) on measures of phonological awareness and letter identification. They found that intervention in phonological awareness and word building had the most impact. Their results also showed that home environment, classroom behavior, phonological awareness, verbal memory, and RAN best predicted progress [For another review of intervention studies and predictions of improvement see Al Otaiba and Fuchs (2002)]. Response to intervention, as a measure for predicting children's reading improvement over time was also found in the study conducted by Speece (2005).

Another study that sought to examine the performance of preschoolers prior to formal instruction, as well as to find possible prediction measures for this age was conducted by Spira et al. (2005). The researchers note that a child's performance on reading ability measures at the end of first grade is, generally, a good indication of their status in later years of schooling (Juel, 1988). That is to say, stabilization of readiness is reached by the end of first grade. Children with high scores in phonological awareness, spoken language, literacy knowledge (knowledge of the alphabet, letter identification, and word identification), in addition to classroom behavior (such as the ability to remain seated in class, as measured by the Conners Test), showed an improved ability later on, in contrast to those children who displayed lower linguistic and behavioral skills.

Another explanation of the apparent differences between children who showed improvement and those who did not also has to do with whether or not the child can rely on other abilities for reading acquisition. These children with encoding difficulties were able to rely on other linguistic resources and managed to attain parity of reading level with their classmates, due to their strong linguistic abilities and appropriate classroom behavior. Some linguistic measures were found to distinguish between children who respond to intervention and those who do not. Denton et al. (2013) found that children who did not respond to intervention displayed difficulties across all linguistic measures including receptive vocabulary, auditory comprehension, phonological awareness, and letter naming (Denton et al., 2013). Stanovich (1980, 1984) and Shaywitz et al. (2003) also showed that difficulty in one reading domain compels readers to rely on information from other sources. The participants who managed to improve, relied on general abilities and linguistic abilities to compensate for weak encoding skills. Siegler's (1997) and Flynn and Rahbar's (1998) findings further strengthen the association between linguistic abilities and written comprehension which increase with reading skill and grade, such that children were assisted by these abilities at higher and more advanced stages of reading. It is evident, based on all the findings described above that it is necessary to be more precise in conclusions made based on cognitive and literacy measures. It is important to take into account other aspects that might affect test results, in order to accurately detect children at risk of reading impairment and to reduce over- and under-identification. Researchers, teachers, and administrators must take into account developmental aspects, student readiness, and previous experiences with early literacy measures before concluding that a child is at-risk of impairment if he or she does not achieve parity with classmates.

To this end, it is necessary to explore whether some children are able to improve their level of reading despite low initial scores on measures, and to investigate whether there are differences between children who managed to achieve reading parity and those who did not.

### The Current Study

The current study examined which children managed to acquire reading in kindergarten (through adult-mediated frontal instruction based on the phonological method), and what characterized the children who did not manage to learn to read in kindergarten. The current study followed the children until the end of first grade. Therefore, we were able to examine which children managed to achieve parity by the end of first grade and acquire reading and those children who retained a gap and difficulties at the end of first grade. In addition, this study will examine the validity of the different predictions by following the children to the end of the first grade. We examined the children's reading ability after one year of schooling (at the end of kindergarten) and their reading ability at the end of the first grade. This information will provide important evidence regarding which kindergarten measures are the most accurate predictors.

The key research questions of the current study were:

1. Do children succeed in learning how to read by the end of kindergarten after a year of instruction?
  - What are the cognitive and linguistic profiles of those children who successfully learn to read as compared to those who did not successfully learn to read in kindergarten?
2. Do the children who did not successfully learn to read in in kindergarten continue to display difficulties or improve their reading abilities by the end of the first grade?
  - What are the cognitive and linguistic profiles of those children who successfully learn to read as compared to those who did not successfully learn to read at the end of the first grade?
  - What are the differences between children who did not successfully learn to read in kindergarten but were able to achieve parity by the end of first grade (the readiness factor) as compared to children who continued to display difficulties (impairment factor)?

## Methods

### Participants

Participants consisted of 98 kindergarteners (boys—*N* = 43, girls—*N* = 55) aged 4.10–6.0 years (mean age 5.27, standard deviation SD = 0.52) from three kindergartens in the national religious school system in Israel. All participants came from a medium-high socio-economic background. No participants were identified as having developmental problems nor visual/auditory impairments. All participants had a typical level of intelligence. Children were only accepted into the study after the investigators received parental consent. All the children learned to read in kindergarten as part of their regular curriculum. Only 93 children participated in the follow-up first grade study due to attrition. Those five children were not included in the last stage of the study.

### Measures

#### Cognitive Measures at the Beginning of Kindergarten

##### Executive Functions (EFs)

*Head-Toes-Knees-Shoulders (HYKS) (Ponitz et al., 2009).* This test assesses the child's ability to regulate his or her behavior, including understanding and following instructions, maintaining attention, inhibition, and flexible thinking. The test is comprised of 20 items. Each item is scored 0 for an incorrect response, 1 for a self-corrected response, and 2 for a correct response. The score range is 0–40. In Part I, the child was asked to place his or her hands on two body parts as instructed by the examiner. In Part II, the child was asked to place his or her hands on two body parts that are the opposite of the instructions given by the examiner (shoulders = toes, head = knees). The child performed the opposite of the dominant response for four different verbal instructions. The internal consistency of this test was 0.90.

##### Memory

*Digit Span (WISC-3R; Wechsler, 1991).* This test examines short-term auditory memory. The child was asked to repeat a series of digits recited aloud by the examiner on an increasing level of difficulty—first, a series of two digits was read out loud, and the number of items in each series increased if the child was successful. The final score was the number of digits the child was able to recall correctly.

*Working Memory—CSOT [Based on McInerney et al. (2005)].* In this test, a list of words was read aloud by the examiner. The child was asked to recall the words according to their size, from the word representing the smallest item to the word representing the largest item. The number of items increased if the child succeeded. The score was comprised of the number of words the child managed to recall correctly according to their size (Crohn-Bach Alpha was 0.82).

*Word Span (Kaufman and Kaufman, 2004).* In this test, the child was asked to point to the shadows of familiar objects in the order in which the examiner called out their names. The examiner began with a series of two objects. The number of objects was increased by one if the child was able to successfully recall all the objects in the previous stage. The score was comprised of the number of words correctly recalled by the child.

*Matrices (Kaufman and Kaufman, 2004).* This test examines short-term visual-spatial span as manifested by the child's ability to repeat a sequence of illustrations by their location on a matrix of squares. The test began with a series of two stimuli in a matrix of 9 squares. If the child was successful, another illustration was added to the matrix in the next stage. The score was comprised of the number of items the child recalled correctly.

##### Visual Perception

*Visual Attention—NEPSY (Korkman et al., 1998).* In this test the child was presented with an A3-sized sheet of paper portraying different types of figures. The child was asked to find and mark the 20 bunnies on the sheet. The task ended when the child said that he or she was finished or after 3 min had elapsed. The score was composed of the number of bunnies identified minus the incorrected marks.

*Visual Perception (Beery et al., 1997).* This test included 30 items which increased in level of difficulty. The child was presented with target stimuli (shapes) that he or she was asked to identify from a given series of shapes, by pointing. The time allotted for the test was 3 min. The test score was calculated by the sum of the correct answers ^*^divided by^*^ the time it took to administer the task if the child made an error or the task was interrupted. The internal consistency of this task was 0.92.

*Speed of Processing—Cross Out (Woodcock and Johnson, 1989).* In this test a page was presented to the child, divided into lines. Each line began with the target stimulus (shape). The child was asked to mark, with a diagonal line, all of the similar shapes in the same line. The time allocated for this test was 3 min. The child was asked to perform the test in the fastest and most accurate manner possible. The score was the number of the correct items marked by the child.

#### Linguistic Measures

##### Language

*Expressive Vocabulary—(Kaufman and Kaufman, 2004).* In this test, a word was said aloud to the child, and the child was asked to respond with a word that had the opposite meaning. The measure was comprised of 14 items ranked dichotomously (correct/incorrect). The score was the number of correct responses.

##### Naming

*Continuous Naming of Objects (Shatil and Share, 2003).* In this test, the child was asked to name 21 pictures of objects as quickly as possible. Naming time was measured in seconds. The child's number of errors on the entire test was recorded. Familiarity with the names of the objects was checked before administering the test (test-retest reliability was *r* = 0.72 for naming speed, and *r* = 0.85 for naming accuracy).

*Naming Single Letters (Schwartz, 2006).* In this test, the child was asked to name 10 letters presented to him or her on cards. Scoring of the task was the number of letters that the child named correctly. The internal consistency of this task was 0.85.

*Orthographic/Word Identification [Based on Shaul (2011)].* This test consisted of 10 items. For each item a target word was read aloud to the child, who was asked to identify it from among four printed words presented to him or her. The distractors presented differed from the target word by one letter, two letters, or all letters. For a correct answer the child received three points, for choosing a distractor with two identical letters to those of the target word two points, for choosing a distractor where the first letter was identical to that of the target word one point, and for choosing a distractor that differed from the target word in all the letters no points. The final score for the task was calculated by summing the points for each of the items. The internal consistency of this task was 0.85.

*Word Writing [Based on Schwartz (2006)].* This test was intended to evaluate the children's ability to write basic Hebrew words. In this task the child was asked to write his or her first name and five other words: *ima* (“mother”); *shalom* (“peace”); *bayit* (“house”); *pil* (“elephant”); and *nemala* (“ant”). The chosen words were all nouns that are common in Hebrew. The words are also from the array used in the study by Levin et al. (1996).

*Isolation of First Phoneme (Schwartz, 2006).* This test examined the child's ability to isolate the first consonant phoneme in a word. For instance, the opening phoneme in the word “banana” is /b/. The child was asked to label 10 pictures and to say the first phoneme he or she heard in the word. For each correct response the child was rewarded with one point (for a maximum of 10 points). The internal reliability of this test was α = 94.

*Omitting a Syllable From a Word (Shani et al., 2006).* In this test, the child was presented with 14 words. The child was then asked to omit a syllable. The omission generated a real word. The percentage of errors was calculated. The internal consistency of this task was 0.79.

#### Reading Tests at the End of Kindergarten (Ministry of Education, 2014)

##### Reading Single Words

This test checked the efficacy of reading and identifying words on measures of speed and accuracy. The number of words the child is able to read in 1 min and the number of words read correctly were recorded. The internal consistency of this task was 0.72.

##### Reading Non-words

This test checked phonological knowledge. The child was instructed to read nonsense words in Hebrew. This test checked the child's ability to decode without using meaning. The number of nonsense words the child read in 1 min and the number of words read correctly were recorded. The internal consistency of this task was 0.85.

##### Reading a Story

This test checked the speed and accuracy of vocal reading of a short text. The child was given an age-appropriate vowelized narrative text and was asked to read it as accurately and rapidly as possible. The number of words the child read per minute and the percentage of errors were recorded.

#### Reading Measures at the End of First Grade (Shani et al., 2006)

##### Reading Single Words

This test checked the efficacy of reading and identifying words on measures of reading accuracy and speed. The child was asked to read 38 vowelized words aloud which represent different levels of frequency, length, and morphological structure. The number of words read correctly per minute and the percentage of errors were recorded. The internal consistency of this task was 0.90.

##### Reading Non-words

This measure tested phonological decoding. In this test, the child was asked to read 33 vowelized nonsense words (that do not exist in Hebrew). The number of words read correctly per minute and the percentage of errors were recorded. The internal consistency of this task was 0.91.

##### Reading a Story

This test checked the speed and accuracy of reading in context. The child was asked to read a vowelized text that was age-appropriate in both content and length. The number of words per minute and the percentage of errors were recorded.

### Research Procedure

The study is a longitudinal study that tested children at three different time points: the beginning of kindergarten, the end of kindergarten, and the end of first grade. The study itself was approved by the Ethics Committee at the University of Haifa and all parents of the participants gave their consent.

The kindergarteners learned to read in kindergarten as part of their regular curriculum. The instruction was primarily individualized, each child received 5–10 min of instruction per day, five days per week, throughout the school year. The children learned the letters systematically, one by one and then began to learn the vowels. The children only began to practice fluent reading by customary practice methods for boosting reading fluency, once they acquired all the vowels.

All the tests were administered to the children individually while in kindergarten and at school. Each child met with the examiner individually in a quiet room with a child-appropriate table and chairs. To prevent tiredness and inattention by the children, tests were administered over several sessions (2–3 sessions each, according to the child's engagement). Each session lasted a maximum of 20 min. The sessions were held at the beginning of kindergarten and at the end of kindergarten, and about one year later at the end of first grade The children were given cognitive and linguistic tests at the beginning and end of the year—after learning to read, reading tests were performed. Additionally, all the children were tested on the reading tests in order to measure their reading development at the end of first grade. Another aim of the testing was to check which children were able to acquire reading at a later point in time and to see which participants were unable to acquire reading by the end of first grade. The timeline of testing is presented below:


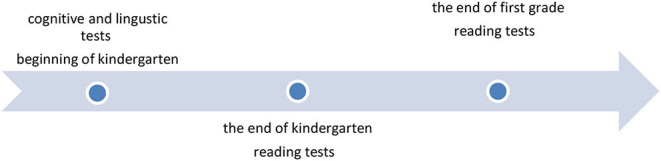


## Results

### Research Question 1: Do Children Successfully Learn How to Read at the End of Kindergarten?

According to the children's reading tests at the end of kindergarten, four different reading profiles were found, with the division following percentile-based cross-sections:

1. **IS** group: Inaccurate and slow readers which included children who performed below the 25th percentile for speed and accuracy (19 children, about 20%).
2. **AS** group: Accurate but slow readers which included children who performed above the 25th percentile on accuracy and below the 25th percentile in speed (9 children, about 9%).
3. **IF** group: Inaccurate but fluent readers which included children who performed above the 25th percentile on speed but below the 25th percentile in accuracy (8 children, about 8%).
4. **AF** group: Accurate and fluent readers which included children who performed above the 25th percentile on speed and accuracy (62 children, about 63%).

**Figure 1:**
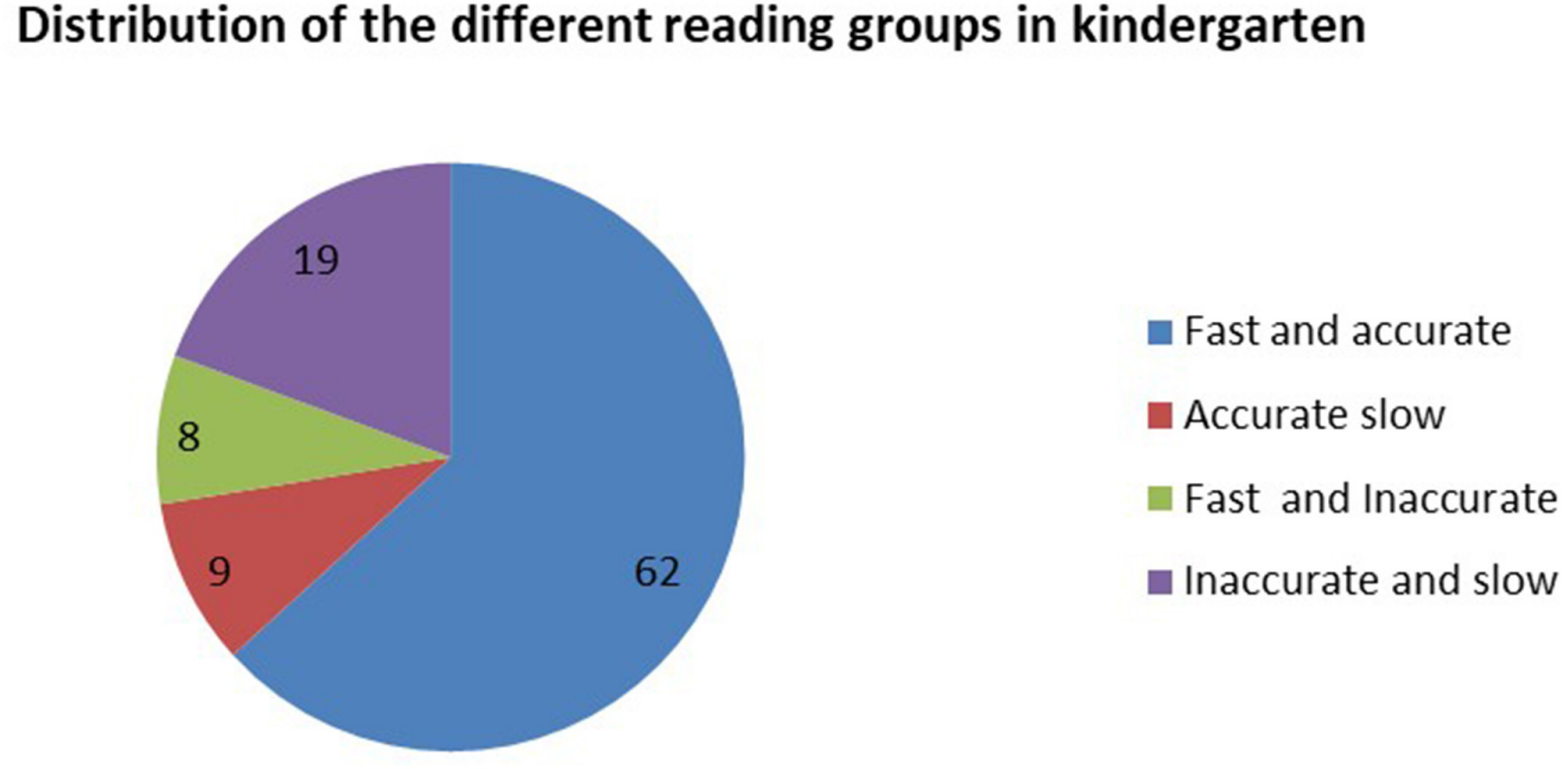
Distribution of children by level of reading in kindergarten.

The groups are presented in Figure 1. It is clear from Figure 1 that most of the children (63%) successfully learned reading accuracy and fluency (AF group). However, 20% of the children were still struggling with both reading speed and accuracy at the end of kindergarten (IS group). An additional 17% of the participants were struggling with either speed or accuracy (i.e., had not yet completed the process of reading acquisition) at the end of kindergarten (IF and AS groups).

**Figure 2:**
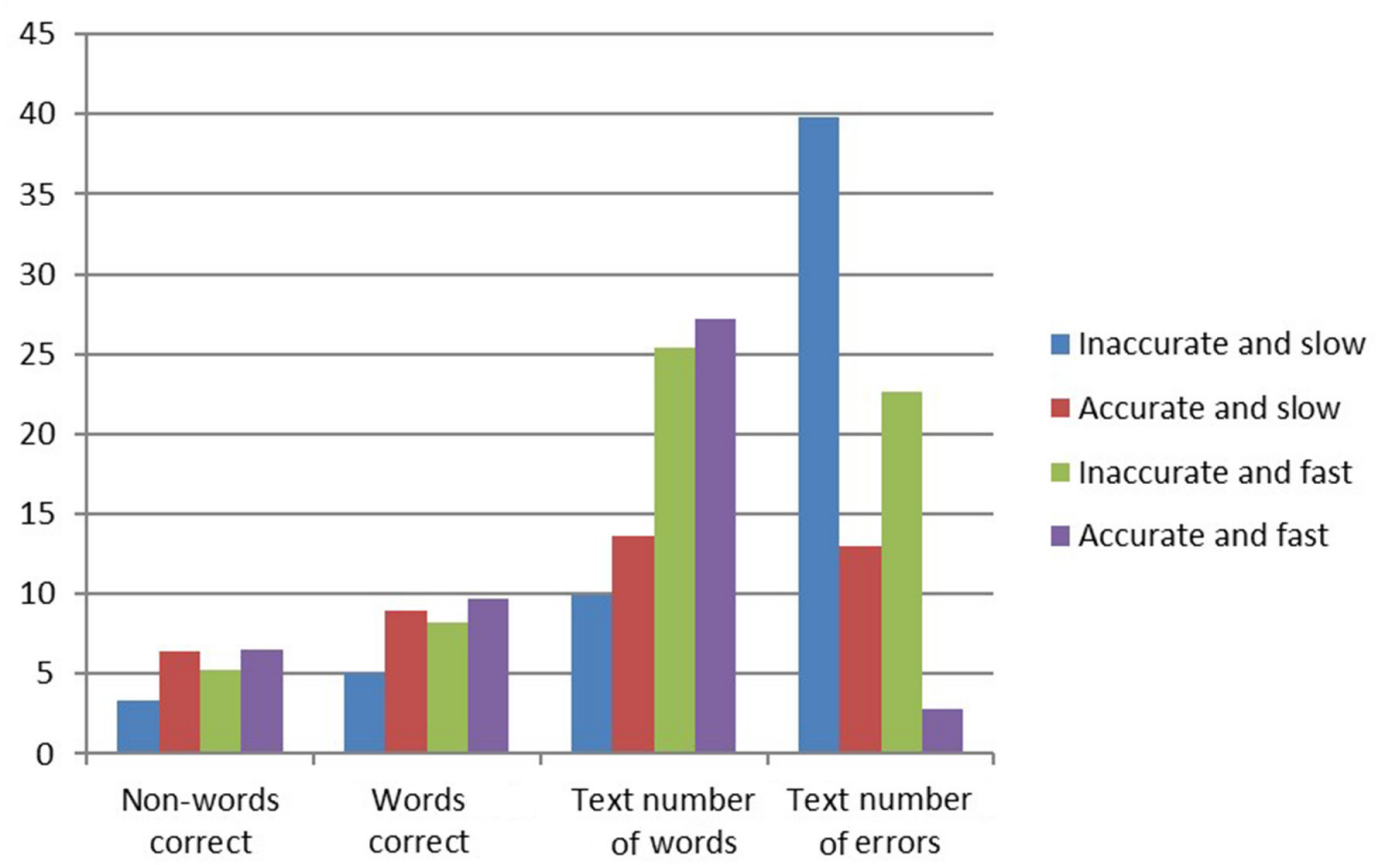
Differences between the groups in reading accuracy at the end of kindergarten.

**Figure 3:**
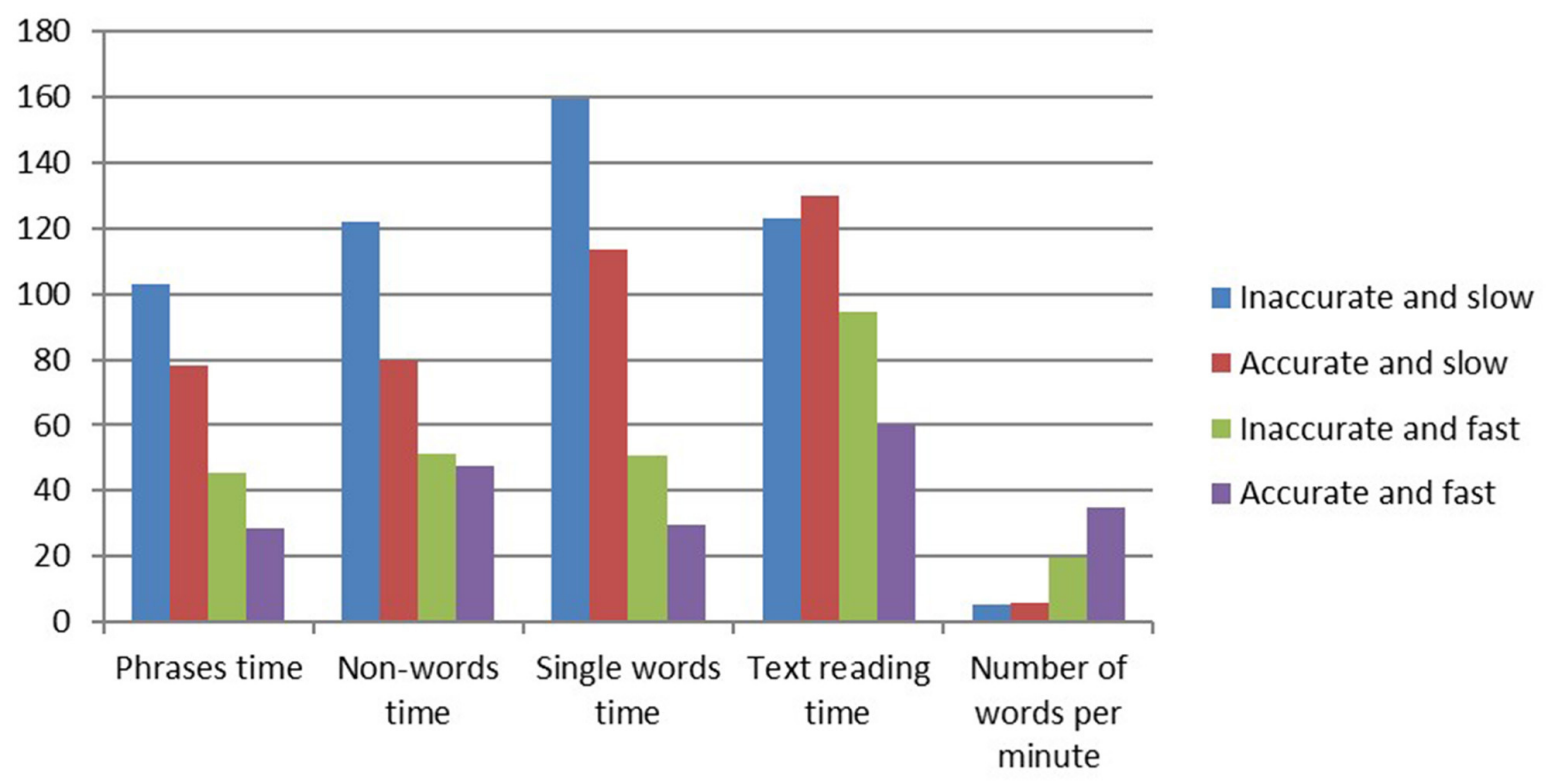
Differences between the reading groups in reading speed at the end of kindergarten.

Table 1 presents the means and standard deviations of the reading measures at the end of kindergarten in each of the four reading groups and further validates the distribution by statistical differences which distinguish the form each other based on the various measures. In addition, the reading profiles of each of the reading groups, regarding accuracy and speed, can be seen in Figures 2, 3. From Figures 2, 3, it is evident that different profiles of reading are distinguishable via reading speed and accuracy. These groups were further found to differ from each other statistically, as evidenced by Table 1.

**Table 1:** Means and standard deviations of the various reading groups, and their differences.

	**Inaccurate and slow** ** (*****N*** **=** **19)** ** Group 1 (IS)**		**Accurate and slow** ** (*****N*** **=** **9)** ** Group 2 (AS)**		**Inaccurate and fast** ** (*****N*** **=** **8)** ** Group 3 (IF)**		**Accurate and fast** ** (*****N*** **=** **62)** ** Group 4 (AF)**		***F*_**(3.97)**_**
	**Mean**	**SD**	**Mean**	**SD**	**Mean**	**SD**	**Mean**	**SD**	
Text—accuracy	13.68	(5.97)	20.00	(1.22)	20.13	(1.72)	21.27	(1.08)	^***^35.32, 1 <3, 1 <2, 1 <4
Text—time	102.93	(41.99)	78.05	(28.16)	45.41	(10.30)	28.50	(10.25)	^***^61.04, 1, 2 <3, 1, 2 <4
Non-words accuracy	3.32	(1.94)	6.44	(0.52)	5.25	(1.28)	6.50	(0.71)	^***^42.50, 1, 3 <2 1 <3, 1, 3 <4
Non-words—time	122.19	(61.59)	79.51	(31.36)	51.36	(34.27)	47.55	(47.75)	^***^51.92, 1, 2, 3 <4 1 <3
Words—accuracy	5.05	(2.41)	9.00	(0.86)	8.25	(1.28)	9.73	(0.65)	^***^67.38, 1, 3 <4 1 <2, 1 <3
Words—time	159.48	(70.89)	113.78	(47.03)	50.53	(24.60)	29.63	(15.65)	^***^65.80, 1, 2 <4 1 <3
Words per minute	5.04	(3.79)	6.07	(2.51)	19.31	(10.62)	35.13	(19.39)	^***^21.91, 1,2,3 <4 1 <3

*** *p <0.001*.

#### The Cognitive and Linguistic Profiles of the Different Reading Groups at the End of Kindergarten

A one-way analysis of variance (ANOVA) followed by Bonferroni *post-hoc* comparisons tests were performed in order to determine the significance of the differences between the different reading groups on the different cognitive and linguistic measures. The findings (mean, SD, and F values) are presented in Tables 2, 3.

**Table 2:** Differences between the various groups at the end of kindergarten on the cognitive measures at the beginning of the year.

	**Inaccurate and slow!!break Group 1 (IS)**		**Accurate and slow!!break Group 2 (AS)**		**Inaccurate and fast!!break Group 3 (IF)**		**Accurate and fast!!break Group 4 (AF)**		***F*_**(3.97)**_**
	**Mean**	**SD**	**Mean**	**SD**	**Mean**	**SD**	**Mean**	**SD**	
Visual perception	13.56	(2.97)	12.89	(3.21)	13.88	(4.29)	14.48	(3.06)	0.93
Working memory	15.39	(4.91)	14.67	(4.5)	14.38	(6.63)	18.21	(5.91)	^*^2.48 1 <4
Rapid naming	33.43	(6.19)	32.27	(4.99)	36.84	(6.28)	33.52	(11.66)	0.33
EF—inhibition	30.00	(5.56)	33.56	(6.06)	29.25	(14.53)	32.35	(6.34)	1.02
Word span	5.56	(2.40)	8.22	(1.92)	5.88	(2.41)	6.84	(2.51)	^*^2.84, 1 < 2, 1 < 4 3 < 2
Speed of processing	27.50	(10.08)	25.11	(10.45)	27.63	(10.50)	31.19	(10.07)	1.45
Spatial memory	6.17	(2.59)	8.89	(2.57)	6.00	(3.20)	7.27	(2.86)	2.34
Digit span	3.17	(1.09)	3.78	(1.30)	3.00	(1.51)	3.56	(1.47)	0.81
Visual attention	106.20	(34.14)	111.10	(26.39)	122.70	(40.42)	103.51	(34.06)	10.81

* *p < 0.05*.

**Table 3:** Differences between the reading groups at the end of kindergarten on the literacy measures in kindergarten at the beginning of the year.

	**Inaccurate and slow** ** Group 1 (IS)**		**Accurate and slow** ** Group 2 (AS)**		**Inaccurate and fast** ** Group 3 (IF)**		**Accurate and fast** ** Group 4 (AF)**		***F*_**(3.97)**_**
	**Mean**	**SD**	**Mean**	**SD**	**Mean**	**SD**	**Mean**	**SD**	
Phoneme isolation	15.68	(7.67)	18.57	(5.94)	13.29	(7.27)	20.12	(8.26)	^*^2.43 1 <4
Syllable omission	1.22	(1.76)	2.67	(2.23)	1.75	(2.71)	3.76	(3.34)	^**^3.97 1 <4
Word writing	7.67	(3.04)	13.75	(8.34)	11.14	(6.51)	16.33	(9.74)	^**^5.41 1 <4
Letter naming	3.05	(2.29)	4.44	(4.03)	5.50	(3.66)	6.34	(3.21)	^**^5.51 1 <4
Word identification	15.39	(4.08)	19.11	(5.77)	18.88	(2.90)	20.02	(5.73)	^*^3.53 1 <4
Vocabulary	6.67	(2.59)	7.56	(2.50)	7.38	(2.06)	8.53	(2.88)	2.40

* *p < 0.05*,

** *p < 0.01*.

Table 2 presents the results of the cognitive measures. It is evident from Table 2 that the measures of memory (working memory and word span) were able to distinguish significantly between the various groups. The AF group which successfully acquired reading, had a higher range of word recall and working memory scores than the children in the IF, AS, and IS groups. In addition, the word span test was able to distinguish between the AS group and the IS and IF groups who displayed inaccurate reading. In the other measures no differences were found between the four reading groups.

The findings (mean, SD, and F values) of the one-way ANOVA analysis of variance of the literacy measures for the different reading groups is presented in Table 3. It is evident from Table 3 that the literacy measures distinguished between the different groups and particularly between the extremes: namely the AF and IS groups. Thus, the AF group, participants who acquired full reading skills, had higher literacy abilities at the beginning of kindergarten and scored higher than the IS group who did not manage to acquire reading at all. This is evident on all the linguistic measures (phonological awareness, orthographic knowledge, and writing abilities) with the exception of vocabulary.

In summary, the majority of the children who learned to read by the end of kindergarten had high scores on both literacy and cognitive measures at the beginning of kindergarten (AF group). Moreover, children who did not learn to read by the end of kindergarten had the lowest literacy scores and memory difficulties at the end of kindergarten (IS group).

### Research Question 2: Do Children Who Displayed Difficulties in Reading at the End of Kindergarten Improve or Continue to Show an Impairment at the End of First Grade?

This question aimed to examine the impact of learning to read in kindergarten on further learning in the first grade, and the possible differences between children who continued to display difficulties in reading acquisition compared to those who achieved reading parity in the first grade. In order to examine this question, reading measures were collected at the end of first grade across all the participants. Children were defined as having difficulty reading if they were below the 25th percentile for reading words and non-words at the end of the first grade.

In order to examine the connection between the reading groups at the end of kindergarten compared to the groups at the end of first grade, a crosstabs analysis was performed. A significant connection was found between the distribution of the groups in kindergarten and in first grade, such that 70% of the children (65 of 93) continued in the same reading group from kindergarten through first grade, (χ^2^ = 48.3, *p* < 0.001). It is important to note that 12 children who could not read at the end of kindergarten improved their reading by the end of first grade, moving into the AF group. The distribution of the groups is presented in Table 4.

**Table 4:** Transition of children between the different reading groups in comparison between the first grade and kindergarten.

	**Reading status in first grade**			
**Reading status in kindergarten**	**Inaccurate and slow (IS)**	**Accurate and slow (AS)**	**Fast and inaccurate (IF)**	**Fast and accurate (AF)**
Inaccurate and slow (19)	7	2	5	5
Accurate and slow (8)	2	3	0	3
Fast and inaccurate (7)	1	1	2	3
Fast and accurate (59)	0	4	2	53
Total	10	10	9	64

#### The Cognitive and Linguistic Profiles of the Reading Groups at the End of First Grade

In order to examine the differences in cognitive and linguistic profiles of the reading groups at the end of first grade, a one-way ANOVA followed by Bonferroni *post-hoc* comparisons tests were performed. The statistical analyses allowed the investigators to determine if there were significant differences between the different reading groups on the initial kindergarten measures. The cognitive and linguistic profiles of the reading groups (at the end of first grade) on the measures collected at the beginning of kindergarten are presented in Tables 5, 6.

**Table 5:** Comparison between the reading profile at the end of first grade and the cognitive profile at the beginning of kindergarten.

**Measures at the beginning of kindergarten**	**Slow and inaccurate** ** Group 1 (IS)**		**Slow and accurate** ** Group 2 (AS)**		**Fast and inaccurate** ** Group 3 (IF)**		**Fast and accurate** ** Group 4 (AF)**		***F*_**(3.92)**_ (differences between the groups)**
	**Mean**	**SD**	**Mean**	**SD**	**Mean**	**SD**	**Mean**	**SD**	
Visual perception	11.10	3.31	15.22	2.38	15.11	2.93	14.42	3.18	^**^3.98 1 <3, 1 <2, 1 <4
Working memory	14.60	5.70	11.78	5.23	15.56	4.61	18.09	5.61	^**^4.36 2 <4
Rapid naming	37.17	6.92	41.82	12.79	31.26	5.67	32.48	9.94	^*^3.07 2 <4
EF- inhibition	25.80	6.16	28.56	12.82	34.44	4.61	32.57	6.07	^*^3.91 1 <3, 1 <4
Word span	4.90	1.79	6.33	2.50	5.33	2.17	7.03	2.52	^*^3.16 1 <4
Speed of processing	22.20	8.13	24.44	6.67	36.22	9.88	30.14	10.16	^**^4.24, 1 <3
Spatial memory	5.20	2.44	8.22	4.02	7.00	3.24	7.32	2.67	2.06
Digit span	2.80	0.91	3.11	1.16	2.44	1.01	3.71	1.46	^*^3.36 3 <4
Visual attention	131.44	29.86	115.88	26.55	81.86	24.58	106.12	34.31	^*^3.92 1 <3

* *p < 0.05*,

** *p < 0.01*.

**Table 6:** The profiles of the different reading groups by the end of first grade on the literacy measures at the beginning of kindergarten.

	**Slow and inaccurate** ** Group 1 (IS)**		**Slow and accurate** ** Group 2 (AS)**		**Fast and inaccurate** ** Group 3 (IF)**		**Fast and accurate** ** Group 4 (AF)**		***F*_**(3.92)**_**
	**Mean**	**SD**	**Mean**	**SD**	**Mean**	**SD**	**Mean**	**SD**	
Phoneme isolation	12.70	7.43	16.20	8.36	14.56	6.02	20.44	7.90	^**^4.26 1 <4
Syllable omission	0.90	1.59	2.67	2.23	1.00	1.50	3.52	3.35	^*^3.64 1, 3 <4
Writing words	8.90	3.51	10.56	4.87	8.11	3.68	16.20	9.89	^**^4.41 1, 3 <4
Letter naming	2.50	2.59	2.60	2.11	4.11	2.57	6.34	3.26	^***^8.41 2, 1 <4
Word identification	15.40	4.62	17.22	6.70	16.89	4.31	20.02	5.43	^*^3.03 1 <4
Vocabulary	6.20	2.57	7.56	3.20	7.11	2.02	8.43	2.84	2.28

* *p < 0.05*,

** *p < 0.01*,

*** *p < 0.001*.

It is evident from Table 5, which presents the mean, SD and F values of the analysis of the cognitive measures, that most of the cognitive measures were found to significantly differentiate between the groups. However, the spatial memory measure did not constitute a distinguishing factor. Visual perception, working memory, visual attention, and inhibition were significantly lower among the IS group, those participants did not successfully learn to read by the end of first grade.

**Figure 4:**
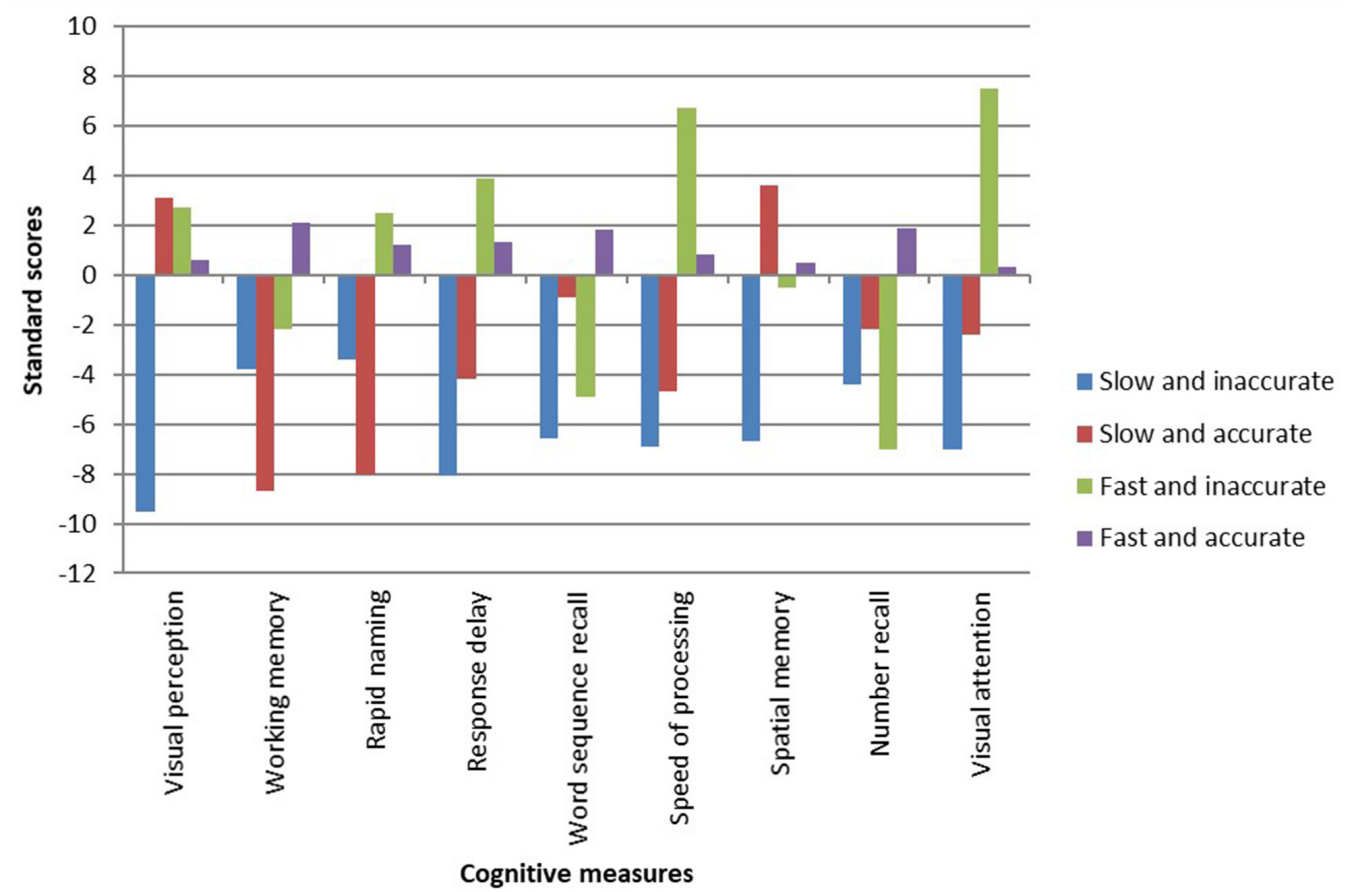
Differences in the cognitive measures (standard scores) in the reading groups (end of first grade).

**Figure 5:**
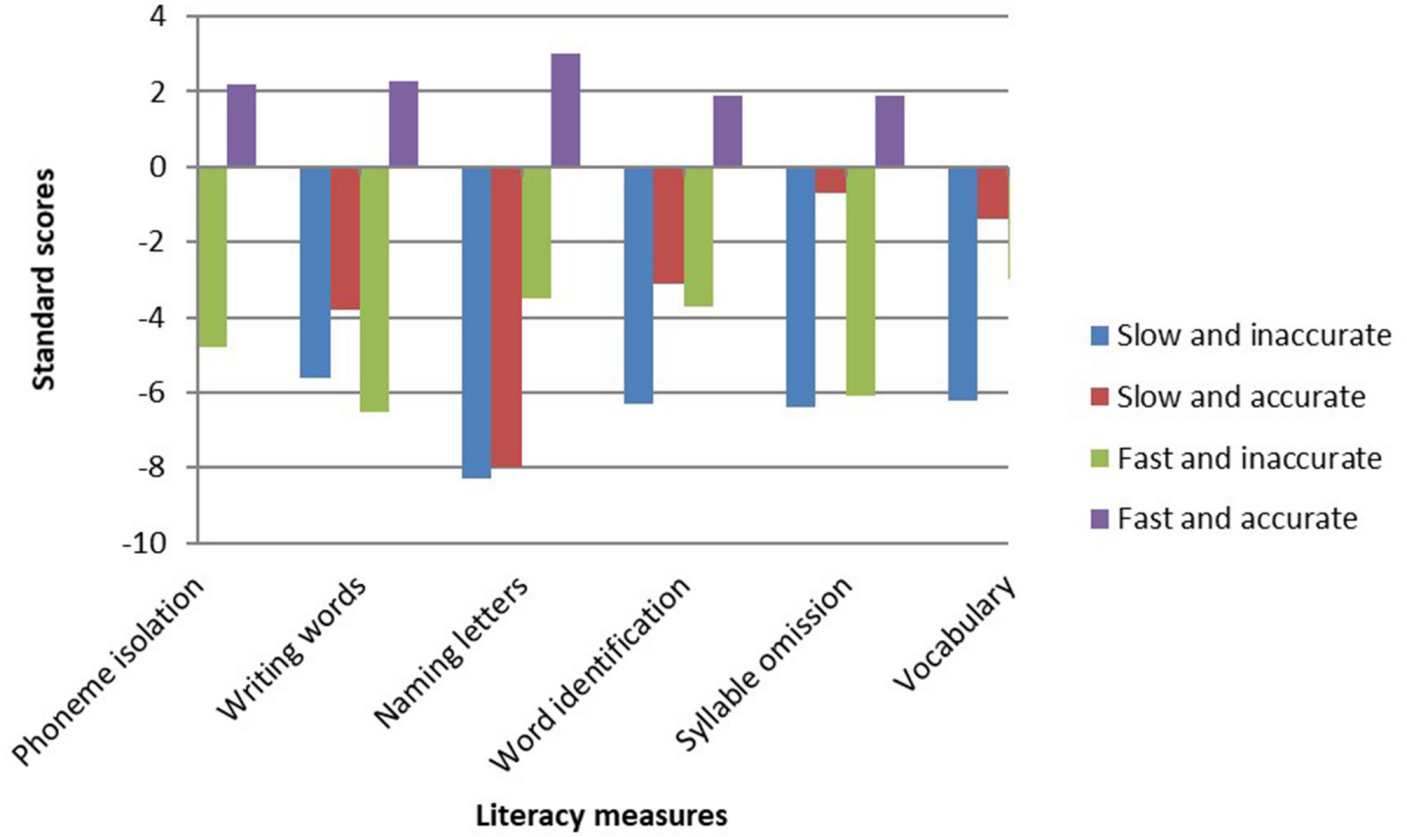
Differences in literacy measures (standard scores) in the reading groups (end of first grade).

The differences between the reading groups on linguistic measures are presented in Table 6. Table 6 clearly shows that all literacy measures distinguished between the four reading groups, except vocabulary. Children who successfully learned to read by the end of first grade performed better on measures of phonological awareness, letter knowledge, and orthographic knowledge than the other reading groups. A closer look at the differences between the different subgroups of reading in the first grade reveals that the AS group had lower processing speed and EF abilities. The AS group had difficulties with other literacy measures, especially letter naming, visual perception, and short-term memory (STM). The IF group had high EF abilities and visual perception scores, but was weak in the literacy and phonological domains. The AF group was also weak in STM. Figures 4, 5 present the profiles of the cognitive and linguistic measures in each of the four groups.

#### The Differences Between the Group That Improved in Reading at the End of First Grade and the Children Who Continued to Show Difficulties

In order to examine the differences between the various groups of children (especially between those who showed improvement and successfully learned to read by the end of first grade vs. those who did not achieve parity) an additional distribution into groups was made by improvement from kindergarten to first grade.

The children were divided into three new groups named based on Vellutino et al. (1996):

1. Very Little Gain (VLG) Group children who continued to display difficulties in first grade.
2. Good Gain (GG) Group children who struggled at the end of kindergarten but improved by the end of first grade.
3. Very Good Gain Group (VGG) children who read well at the end of kindergarten and maintained this ability at the end of first grade.

In order to investigate the difference between these three groups, a one-way ANOVA followed by Bonferroni *post-hoc* comparisons tests were utilized. A *t*-test was also conducted in order to examine the differences between the VLG and GG groups; those who did not manage to achieve reading parity and those who managed to improve. The findings of the analyses of the cognitive and linguistic measures (mean, SD, F, and T values) are presented in Table 7.

**Table 7:** Literacy and cognitive measures descriptive of the three groups and the F and T values of the differences between them.

**Cognitive and linguistic measures in kindergarten**	**Did not do well in kindergarten and remained challenged** ** Group 1 (VLG)** ***N*** **=** **7**		**Did not manage to learn in k. but improved in 1st grade** ** Group 2 (GG)** ***N*** **=** **11**		**Managed to acquire reading and remained strong** ** Group 3 (VGG)** ***N*** **=** **53**		**F Differences between groups**		***T*-test Groups 1-2**
	**Mean**	**SD**	**Mean**	**SD**	**Mean**	**SD**	**F_**(2.70)**_**	***Post-hoc***	
Letter naming	2.50	2.59	6.00	3.60	6.38	3.24	6.11^**^	1 <3, 1 <2	^*^2.53–
Word identification	15.40	4.62	17.55	4.27	20.51	5.58	4.67^*^	1 <3	1.10–
Syllable omission	0.90	1.59	2.00	2.19	3.81	3.50	4.42^*^	1 <3	1.30–
Phoneme isolation	12.70	7.43	20.50	4.47	20.55	8.44	4.22^*^	1 <3	^*^2.04–
Writing words	8.90	3.51	11.27	8.63	17.09	9.96	4.46^*^	1 <3	0.80–
Vocabulary	6.20	2.57	7.27	2.76	8.66	2.85	3.82^*^	1 <3	0.91–
Visual perception	11.10	3.31	14.00	3.19	14.40	3.14	4.54^*^	1 <3	^*^2.04–
Working memory	14.60	5.70	15.45	3.98	18.62	5.83	3.13^*^	1 <3	0.40–
Rapid naming	37.17	6.92	32.25	4.74	32.64	10.79	0.97		1.91
Inhibition	25.80	6.16	33.45	5.52	32.34	6.26	5.32^**^	1 <3, 1 <2	^**^3.00–
Word span	4.90	1.79	7.91	2.38	6.89	2.54	4.19^*^	1 <2	^**^3.24–
Speed of processing	22.20	8.13	26.00	10.78	30.77	9.90	3.76^*^	1 <3	0.90–
Spatial memory	5.20	2.44	6.27	1.73	7.47	2.77	3.66^*^	1 <3	1.16–
Digit span	2.80	0.91	4.09	1.30	3.60	1.49	2.25		^*^−2.60
Visual attention	131.44	29.86	108.87	37.76	105.51	34.25	2.41		1.50

* *p < 0.05*,

** *p < 0.01*.

Table 7 shows that significant differences were found between the groups on all literacy measures. Most notably, the VGG group (strong and remained strong) did significantly better than VLG group (continued to show difficulties). In addition, the GG group (improved during the first grade) had higher accuracy on the letter naming task than the VLG group. With regard to the cognitive measures, the VGG group performed better than the VLG group on measures of visual perception, working memory, processing speed, and spatial memory. In addition, EF and word span (memory) measures were found to significantly distinguish between the GG and VLG groups.

Overall, the GG group achieved significantly better scores than the group who continued to have difficulties on these measures. The *T*-test analysis between the two groups (the children who improved compared to the children who continued to show difficulties) showed that, in addition to the measures were found to differ significantly between the three groups in the one-way ANOVA, the measures of phoneme isolation, visual perception, and digit span (short term memory). The children who improved showed better performance on all of these measures.

## Discussion

This study explored the process of learning to read in kindergarten. Specifically, the investigators examined the cognitive and linguistic profiles of children at the beginning of kindergarten, and how these profiles affected the process of learning to read at the end kindergarten and at the end of first grade. The purpose of the study was to explore the efficacy of teaching reading in kindergarten and whether it is possible to find cognitive and linguistic abilities at the beginning of kindergarten that distinguish between different groups of readers at the end of kindergarten. The study found that most of the children who learned to read in kindergarten managed to acquire the skills well. Moreover, specific features were found for the different reading ability groups of children. Specifically, those children who managed to learn how to read in kindergarten and continued to show difficulties in first grade(VLG) performed the lowest on most cognitive and linguistic measures.

Previous research evidence indicates that the closer the study is to the age at which children begin reading the higher its predictive validity (Ellis and Large, 1988; Lonigan and Shanahan, 2009). Therefore, the study examined children who began to learn to read in kindergarten. The age of the participants made it possible to more accurately detect differences between inability to learn to read due to a fundamental difficulty with reading, and difficulty with reading due to un-readiness or a lack of experience. It is possible that the temporal proximity of the initial measures to the onset of reading instruction made it possible to more precisely gauge which measures have the best prediction capacity.

### Do Children Successfully Learn to Read in Kindergarten?

Our first research question asked whether, from a cognitive and literacy perspective, children successfully acquired reading at the end of kindergarten, what characterized the children who learned to read, and what characterized those who did not learn to read. The researchers hypothesized that most of the children with high initial literacy and cognitive scores would successfully acquire reading in kindergarten. This hypothesis was confirmed. Therefore, the participants were classified as belonging to one of four reading groups: the two extreme groups (AF) those who managed to fully acquire reading and who read rapidly and accurately, (IS) the group of children who did not manage to learn to read while in kindergarten and read slowly and inaccurately; and the two middle groups that managed to acquire only some of the reading skills—those who managed to read rapidly (IF) but with errors (inaccurately) and those (AS) that managed to read accurately but slowly. This division into groups was based on the study conducted by Shany and Share (2011), among other things. The different profiles are distinguished by the separate mechanisms that generate the different groups such that those who display difficulties in the phonological domain and in morphological knowledge (IS) show inaccurate reading and encoding difficulties while those who display difficulties with speed of processing and rapid naming will display a slow but possibly accurate reading profile (AS) [an example of additional reading profiles can be found in Torppa et al. (2007)].

### The Cognitive and Linguistic Profiles of the Different Reading Groups in Kindergarten

The current study highlights different measures that may be capable of distinguishing between diverse reading groups. The literacy measures, working memory, and digit span were found to distinguish best between the groups. Most interestingly, the digit span measure was able to distinguish between all the four reading groups (IS, IF, AS, and AF). In contrast, the literacy and working memory measures were only able to distinguish between IS and AF groups. This finding reinforces the findings of previous studies, which demonstrated that in the absence of linguistic and cognitive foundations, children struggle to successfully acquiring reading (Facoetti et al., 2010; Nevo and Breznitz, 2011, 2013; Caravolas et al., 2012; Carroll et al., 2014; Partanen and Siegel, 2014). Another interesting finding is that vocabulary measures were unable to distinguish between the different reading groups. Previous research contains a debate about the association between vocabulary and reading. Links have been found between vocabulary and reading comprehension and between vocabulary and reading ability (Ouellette, 2006). A possible explanation for these results is that Hebrew vocabulary contributes to reading only at a later stage of reading comprehension (not the decoding stage) (Whitehurst and Lonigan, 1998). Adams (1990), suggested that, rather than a child's vocabulary, phonological and orthographic (knowledge of print) skills are the foundation of the reading acquisition process. This claim is further supported in the literature by the apparent differences between languages with shallow and deep orthographies. It appears that in languages with shallow orthographies (wherein beginning readers rely on a letter-sound correspondence, like in Hebrew) the association between reading acquisition and vocabulary is decreased. It is possible that the association with vocabulary increases in the more advanced stages of reading—in the whole word reading and reading in context stages. In contrast, emerging readers of languages with deep orthographies need larger vocabularies at the early stages of reading acquisition (Suggate et al., 2014). Another interesting conclusion is that the word sequence span (verbal-visual memory) measure was found to be the only significant measure to distinguish between almost all the groups, including the IF and AS groups which did not fully acquire reading. In this test, the children heard a sequence of nouns and were supposed to point to the pictures that illustrated them in the order in which they were heard. This ability is similar to reading insofar as it connects verbal and visual and follows a sequence of sounds and letters that create a word. According to previous research, Digit Span, which represent short term verbal memory ability (STM), is significant for identifying children at-risk for reading difficulties (Bishop and League, 2006). Use of this test, side by side with standard phonological tests, can make identification of at-risk readers more precise, since Digit Span, was found to distinguish between the reading groups. We found that that those children who managed to remember a larger number of items in the correct sequence (high STM) were more capable of absorbing and remembering word sequences when attempting to read. This has possible implications for additional components of future reading interventions. Further studies are necessary in order to investigate if this is a two-way association. That is, whether practice with sequence memory tasks could also improve the ability to accurately and correctly identify words. It is important to note that the rapid naming (RAN) measure, which was previously found to be a strong predictor of reading and therefore capable of predicting reading success or failure (Wolf and Bowers, 1999; Schatschneider and Torgesen, 2004; Georgiou et al., 2008; Norton and Wolf, 2012; Warmington and Hulme, 2012; Partanen and Siegel, 2014), was not found to be a consistent measure for distinguishing between the different reading groups in kindergarten. This measure was able to distinguish between different reading abilities at the end of first grade. Unfortunately, it was unable to differentiate between children who showed improvement and those who still struggled with reading at the end of first grade. Another explanation, which compliments those of previous research on reading acquisition, has to do with the difference between written languages. Most of the studies conducted on RAN were in English, a language with a deep orthography. In contrast, vowelized Hebrew (which is similar to German, Spanish, and Greek) makes it possible to rely on grapheme-to-phoneme conversion and to acquire the rules of reading as early as the first year of learning. In contrast, English has a deep orthograpy wherein whole orthographic patterns are learned from the outset (Seymour et al., 2003). The RAN test may constitute a measure for “sight word” reading, as learned in English. But the efficacy of RAN as a predictor may be lower in phonetic Hebrew due to the reliance on phonology. Support for this claim can be found in a study that examined the efficacy of the RAN measure for predicting reading abilities in different languages, where this measure was found to be less significant in languages with shallow orthographies (Ziegler et al., 2010). Further research is needed in order to confirm this.

#### Do Children Who Displayed Difficulties in Reading at the End of Kindergarten Improve by the End of First Grade?

Our second research question examined whether reading status at the end of kindergarten remained stable at the end of first grade. We further asked that if changes were evident, what were the cognitive and linguistic differences between those who continued to struggle at the end of first grade and those who struggled only in kindergarten. Namely, this question explores whether there are different profiles of children who are not ready to read by the end of kindergarten but manage to do so in the first grade (un-readiness), vs. children who continue to struggle at the end of first grade (impairment). We hypothesized that some of the children who did not acquire reading in kindergarten would show an improvement in first grade, while others would still display difficulties. We further hypothesized that children who showed an improvement in first grade would present a different cognitive and linguistic profile than those who continued to struggle both at the end of kindergarten and at the end of first grade. This hypothesis was also confirmed. For the majority of participants, reading status in first grade was similar to his or her status at the end of kindergarten. However, some of the children fluctuated between the different groups at the end of kindergarten. Some of the participants who were both inaccurate and slow (IS) readers at the end of kindergarten were found to be either accurate (AS) or rapid readers (IF), or even accurate and fast readers (AF) at the end of first grade. Some of those participants who were in the AF reading group at the end of kindergarten moved to the IS group of readers at the end of first grade. This finding may indicate that at the end of kindergarten these children were in the middle of the reading acquisition process; they acquired some reading skills but needed additional practice to cement the process. Similar variations were evident in the other groups. These variabilities suggest that the process of learning to read continues after the conclusion of formal instruction and that different skills develop at different stages of reading acquisition.

### The Cognitive and Linguistic Profiles of the Different Reading Groups at the End of First Grade

We compared the participants' reading profiles (made up of cognitive and literary measures) at the end of first grade and at the beginning of kindergarten (unrelated to reading ability at the end of kindergarten). The results of this analysis showed that the AF group consisted of children who acquired age-appropriate reading by the end of first grade. The AF participants displayed above-average cognitive and linguistic abilities. IS readers, in contrast, scored below- average on all linguistic and cognitive measures. The two middle groups (IF and AS) displayed deficiencies in language and literacy but showed good achievements in the visual domains. The AS group displayed poor working memory and executive functions, in addition to lower digit and word span results which measured memory. In contrast, the IF group scored well in speed of processing and executive functions, but displayed weakness on the STM (digit span) measure. We believe that the AS group had low literacy and executive functions (the latter of which affected working memory and perhaps visual attention) but strong visual perception.

**Figure 6:**
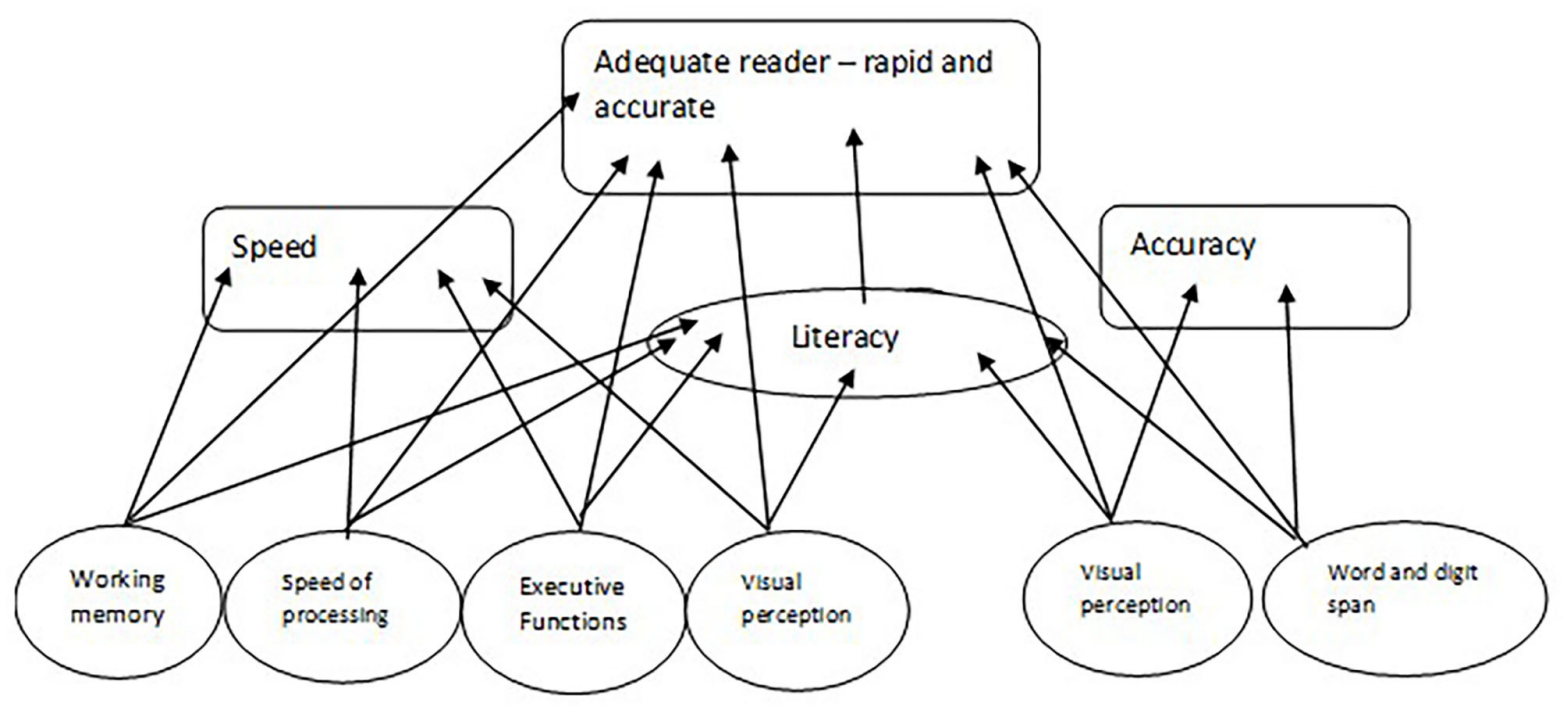
A model which describes the various linguistic and cognitive abilities which influence the reading skills as evident from the study.

In addition to high visual perception, the IF group also had good executive functioning. Like the AS group, they also displayed a weakness in the language domain. The word span measure was found to distinguish between the groups with only with marginal significance. In the AS group, word span appeared to be a strength, in contrast to the IF group whose word span scores were found to be weaker. These results raise the possibility that EF measures are associated with reading speed. That is to say, high EF scores indicate rapid reading and low EF scores indicate slow reading. We further suggest that memory, via the word span measure, is associated with accuracy. The combination of these different domains generates the participant's specific reader profile, as suggested by the model presented in Figure 6. This model shows the links amongst the various mechanisms related to reading acquisition. According to the figure, the most fundamental factors of reading are cognitive abilities. Literacy skills are constructed on the basis of these abilities. The combination of the cognitive and literacy factors forms the profile of the AF reader. From the figure, it is also evident that there are contradictive factors which support different reading profiles. For example, EF mechanisms support reading fluency while STM supports reading accuracy.

Our results also point to the importance visual attention. The data showed that at the end of first grade the IS group performed the visual attention task the most slowly. In contrast, the IF group performed the task most rapidly. These observations may indicate that visual attention in the form of scanning has a strong influence on children's reading outcomes. The IS group scored poorly on this and other tasks. Though the IF group preformed the scanning task very quickly, the speed was not to their benefit as the rapidity of their results led to inaccurate reading. It appears that in order to read well it is necessary to synchronize speed and accuracy and combine them with various other skills (Adams, 1990; Breznitz, 2008).

#### The Differences Between the Participants Who Showed Improved Reading at the End of First Grade and to the Children Who Continued to Have Difficulties

In order to examine the differences between the children who successfully improved reading during first grade and those who continued to struggle, we compared the children's performance on the measures at the beginning of kindergarten, from a cognitive and literacy perspective. Children who continued to struggle at the end of first grade showed low scores at the beginning of kindergarten including both literacy and cognitive measures of memory and EF. In contrast, those whose reading improved during first grade had strong EF scores (inhibition) and good STM abilities. High STM and EF scores seem to have helped these participants to acquire reading, despite a more challenging starting point and initial difficulties in reading acquisition. These mechanisms compensated for the low working memory and for some of the low literacy measures in kindergarten. The children who improved their reading also displayed intra-functional gaps with memory and literacy measures.

Gaps were found in various memory domains like working memory, found to be low, vs. good short-term memory for words. This gap may stem from the specific child's memory strategies. That is, some children have not yet been exposed to reading and have no concept of the phonological dimension. Unlike children and adults who already have reading and phonological awareness and therefore encode information using phonological knowledge (Goswami, 2008), these children were found to utilize visual processing skills to store information. Phonetical knowledge is is also active in working memory mechanisms (Baddeley, 2000). The word span test contains pictures that must be named and remembered. Children at this age may utilize the visual dimension in order to remember the sequence of pictures and therefore are able to successfully perform this task. In contrast to the word span test, the working memory task was auditory-verbal. The differences in the recall strategies at this age may be the reason for the gaps between the different groups. Additional gaps were found on the literacy measures. For example, participants with good phoneme isolation and letter naming abilities were sometimes found to be deficient in writing and word identification abilities. These differences may show that it is necessary to divide the literacy measures into two groups: the logographic (orthographic) parts, which includes word identification and writing words; and the phonological part which includes abilities of phonological awareness and segmentation. The discrepancies we found may attest to insufficient development of fundamental mechanisms.

Thus, based on the model proposed by Frith (1985), some children may have begun to acquire the alphabetic stage but have not yet acquired the logographic stage. Success in phonological tasks constitutes a foundation for acquiring reading, links letters and sounds, and may predict success in reading, as evidenced by the literature (Badian, 2001; Kirby et al., 2003; Frijters et al., 2011; Norton and Wolf, 2012; Warmington and Hulme, 2012). This phonological foundation has been shown to be a developing process with complexity and variety by task (Liberman and Shankweiler, 1985; Adams, 1990). The current study demonstrates the complexity of these tasks. Therefore, we found that the phoneme isolation and letter naming tasks, which require phonological knowledge, were performed well by the children who showed improvement during first grade, in contrast with the syllable omission/segmentation task, on which they scored low. While these children may have adequate fundamental mechanisms; complexity, un-readiness, and the developmental process are manifested in complex tasks such as syllabic omission which also requires a well-developed working memory. These merely adequate mechanisms in kindergarten, likely allowed those children who acquired reading during first grade to compensate for other mechanisms which signaled un-readiness. In contrast, children who stayed in the IS group through the end of first grade likely lacked these mechanisms of support. A possible explanation is that children can rely on other strengths for early reading; Children who initially display difficulty with encoding and reading speed are able to improve their reading abilities over time by relying on their improved skills and the readiness of their linguistic abilities and their general good learning ability (Spira et al., 2005). Shaywitz et al. (2003) support this conclusion as their research showed that a difficulty in one domain of reading compels readers to rely on information from other sources. The children who showed improvement enhanced their linguistic and other abilities in order to compensate for initially deficient encoding skills.

## Conclusions

The findings of the current study showed demonstrable differences in literacy and cognitive skills amongst different reading groups. This study further demonstrated that these differences create a reading profile that can be identified as early as kindergarten. Therefore, a model was proposed which shows that the foundation for learning to read needs both cognitive abilities and literacy skills. A combination of different literacy skills and cognitive abilities represents the profile of an accurate and fluent reader. A deficit in any one of these fundamental mechanisms will generate different reading profiles that will affect accuracy and/or speed.

The study also found that it is possible to distinguish between children who are not ready to read and those who will continue to struggle with reading after foundational skills have been acquired. This study shows that children who to struggle with reading at the end of first grade have deficiencies in both cognitive and literacy domains. In contrast, those children who had sub-standard reading profiles at the end of kindergarten but achieved reading parity by the end of first grade, were not ready to read. They presented a distinct profile in kindergarten: good executive skills combined with intra-functional gaps in memory domains (difficulties with working memory and success in short term memory) as well as gaps in the literacy domains (difficulty with phonological manipulation and logographic identification of spelling patterns vs. success in isolating phonemes and naming letters).

## Implications and Future Research

The results of this study have theoretical and practical implications. Our results will help accurately detect at-risk children in preschool and decrease over generalization due to emphasizing readiness as a distinct predictor. EF plays an important role in distinguishing between types of readers, as well as the verbal word span measure which further facilitated these distinctions. Based on these results, children can be identified and provided intervention at even earlier stages, before beginning formal instruction. Future interventions can be developed that will also enhance executive functions and verbal-visual memory, in addition to early literacy skills like phonological awareness. These future intervention programs will help build and improve the skills needed for reading acquisition. Unfortunately, this study did not examine emotional and motor factors that are related to reading. Further research must take these factors into account. Finally, the current study revealed that a group of children managed to acquire reading in kindergarten, but their reading abilities declined by the end of first grade. It is necessary to continue to investigate the possible reasons for the changing performance of this sub-group.

## Data Availability Statement

The raw data supporting the conclusions of this article will be made available by the authors, without undue reservation.

## Ethics Statement

The studies involving human participants were reviewed and approved by Ethics committee of the faculty of Education university of Haifa. Written informed consent to participate in this study was provided by the participants' legal guardian/next of kin.

## Author Contributions

AN and SS conceptualized this study and contributed to the writing and interpretation of the data. AN contributed to data collection and SS performed the statistical analysis. The writing the first draft was performed by AN. All authors have agreed to be accountable for the content of the work.

## Conflict of Interest

The authors declare that the research was conducted in the absence of any commercial or financial relationships that could be construed as a potential conflict of interest.
